# Metabolome Shift in *Centella asiatica* Leaves Induced by the Novel Plant Growth-Promoting Rhizobacterium, *Priestia megaterium* HyangYak-01

**DOI:** 10.3390/plants13182636

**Published:** 2024-09-21

**Authors:** Min-Chul Kim, HyungWoo Jo, Kyeongmo Lim, Ikwhan Kim, Hye-Been Kim, Sol Kim, Younhwa Nho, Misun Kim, Hyeyoun Kim, Chaeyun Baek, Young Mok Heo, Haeun Lee, Seunghyun Kang, Dong-Geol Lee, Kyudong Han, Jae-Ho Shin

**Affiliations:** 1 Department of Applied Biosciences, Kyungpook National University, Daehak-ro 80, Daegu 41566, Republic of Korea; mckim@mabals.com (M.-C.K.); rudah8668@gmail.com (K.L.); ikwhankim0926@gmail.com (I.K.); 2 MICROBALANCE Co., Ltd., IT Convergence Industrial Building 506, Daehak-ro 80, Daegu 41566, Republic of Korea; 3 R&I Center, COSMAX BTI, Seongnam 13486, Republic of Korea; chohw@cosmax.com (H.J.); kimhb@cosmax.com (H.-B.K.); solkim@cosmax.com (S.K.); yhno@cosmax.com (Y.N.); kimms@cosmax.com (M.K.); hyeyoun@cosmax.com (H.K.); cybaek@cosmax.com (C.B.); ymheo@cosmax.com (Y.M.H.); haeun.lee@cosmax.com (H.L.); shyunk@cosmax.com (S.K.); 4 Department of Microbiology, Dankook University, Cheonan 31116, Republic of Korea; 5 Department of Integrative Biology, Kyungpook National University, Daehak-ro 80, Daegu 41566, Republic of Korea; 6 NGS Core Facility, Kyungpook National University, Daehak-ro 80, Daegu 41566, Republic of Korea

**Keywords:** *Centella asiatica*, *Priestia megaterium*, cosmetics, pharmaceuticals, plant growth-promoting rhizobacterium (PGPR)

## Abstract

*Centella asiatica*, a traditional herb, is widely recognized for its pharmacologically active components, such as asiaticoside, madecassoside, asiatic acid, and madecassic acid. These components render it a highly sought-after ingredient in various industries, including cosmetics and pharmaceuticals. This study aimed to enhance the production and activity of these pharmacological constituents of *C. asiatica* using the plant growth-promoting rhizobacterium *Priestia megaterium* HyangYak-01 during its cultivation. To achieve this goal, the researchers conducted field experiments, which revealed an increase in the production of pharmacologically active compounds in *C. asiatica* cultivated with a *P. megaterium* HyangYak-01 culture solution. Additionally, quadrupole time-of-flight mass spectrometry (Q-TOF MS) confirmed that the composition ratios of the *C. asiatica* extract treated with the *P. megaterium* HyangYak-01 culture solution differed from those of the untreated control and type strain-treated groups. Skin cell experiments indicated that the *C. asiatica* extract treated with the *P. megaterium* HyangYak-01 culture solution exhibited greater skin barrier improvement and less pronounced inflammatory responses than those from plants grown without the bacterial culture solution. This study demonstrates that microbial treatment during plant cultivation can beneficially influence the production of pharmacological constituents, suggesting a valuable approach toward enhancing the therapeutic properties of plants.

## 1. Introduction

*Centella asiatica* is gaining attention across diverse industries, including cosmetics and pharmaceuticals, for its valuable properties. Compounds produced by this plant, such as asiaticoside, madecassoside, asiatic acid, and madecassic acid, possess skin-healing, anti-inflammatory, and antioxidant characteristics [1,2]. In the cosmetic industry, these properties are harnessed to treat wrinkles, promote skin regeneration, improve the skin’s barrier function, and treat eczema and acne. Moreover, in the pharmaceutical field, *C. asiatica* extracts are gaining interest owing to research suggesting their potential benefits in wound healing, enhancing brain function, and promoting cardiovascular health [3]. In the food industry, *C. asiatica* is also incorporated into health supplements and beverages for its advantages in alleviating stress and boosting the immune system [4,5]. The widespread use of this plant underscores its position as one of nature’s most valuable resources, with its popularity further increasing alongside the demand for sustainable sourcing and natural ingredients.

In response to the growing demand driven by interest in the medicinal properties of *C. asiatica*, farmers need to increase production yields. While the extensive use of chemical fertilizers and pesticides is a common method to boost yields in most crops, these conventional farming practices present significant challenges to sustainable cultivation [6,7]. These practices have far-reaching environmental and health implications that warrant careful consideration.

The use of synthetic fertilizers composed of nitrogen and phosphorus can enhance the growth and yield of *C. asiatica*, as well as increase the production of bioactive compounds such as asiaticoside and asiatic acid [8,9]. However, these types of synthetic fertilizers have been identified as a major source of water pollution. When excessively present in agricultural runoff, these nutrients can lead to the eutrophication of aquatic ecosystems, resulting in decreased dissolved oxygen levels and promoting excessive algal growth [10,11]. This phenomenon not only disrupts aquatic ecosystems but also affects water quality in rivers, lakes, and groundwater reserves, posing direct threats to human health [12].

The environmental impact of synthetic agrochemicals extends beyond water pollution. Pesticides can persist in the environment, bioaccumulate in food chains, and adversely affect non-target organisms, including beneficial insects and soil microbiota essential for maintaining soil health [13]. In the context of *C. asiatica* cultivation, the use of such chemicals is particularly concerning due to the plant’s application in herbal medicine, where chemical residues could potentially interfere with its therapeutic efficacy or introduce undesired compounds into herbal preparations [14].

The cumulative effects of these agricultural practices raise concerns about long-term environmental sustainability and the viability of agricultural systems. As awareness of these issues grows, there is an increasing imperative to adopt more sustainable agricultural practices, particularly for the cultivation of medicinal plants like *C. asiatica*. This paradigm shift necessitates the exploration and implementation of alternative methods that can maintain or enhance crop yields while minimizing the environmental impact and ensuring product safety and quality [15]. Such approaches are crucial for the sustainable production of *C. asiatica* and other high-value medicinal crops in the face of growing global demand and environmental challenges.

The overuse of synthetic chemical pesticides significantly impacts soil biodiversity and health [13,14]. Research indicates that pesticides adversely affect non-target organisms, such as earthworms, ants, beetles, and ground-nesting bees, which are crucial for soil health [14]. Moreover, pesticides negatively influence soil microbes that play vital roles in decomposing organic matter and cycling nutrients [15]. These organisms contribute to essential ecosystem services, including nutrient cycling, dead plant and animal decomposition, and pest and disease regulation [16,17]. The altered soil environment resulting from the application of synthetic agrochemicals is both directly and indirectly correlated with plant productivity. In the case of herbaceous medicinal plants such as *C. asiatica*, these modifications can significantly impact the biosynthesis and accumulation of bioactive compounds. Therefore, restricting the misuse of synthetic pesticides and employing formulations that can be used in organic agriculture ultimately benefit the environment and crop production in the long run [18].

Moreover, microbes serve a crucial role in suppressing disease by acting as natural biocontrol agents, competing with harmful organisms, and producing antimicrobial compounds [19,20]. They can directly support plant growth by generating growth-promoting substances and assisting in nutrient uptake [21]. Certain microbes, via symbiotic relationships, enhance nutrient absorption by plants and improve their resilience to environmental stresses like drought [22,23].

In sustainable agricultural systems, practices such as crop rotation, intercropping, conservation tillage, and green manuring are integrated, all profiting from microbial activity [24]. These practices help conserve natural resources, minimize environmental pollution, and maintain soil health and biodiversity. Crop rotation, for instance, aids in nutrient cycling and organic acid decomposition [25], while intercropping potentially improves soil conditions and amplifies crop productivity by effectively utilizing available environmental resources [26]. Conservation tillage alters the spatial distribution of soil microbes, promoting soil organic matter retention, and green manuring incorporates green plants into the soil, enhancing nutrient supply to subsequent crops [27]. By embracing the use of microbes in agriculture, farmers can work toward more sustainable and efficient farming methods that support not only current but also future generations. For medicinal herbs like *C. asiatica*, these sustainable practices can significantly influence both biomass yield and the production of bioactive compounds. The enhanced soil microbial diversity and improved nutrient cycling resulting from these methods may potentially optimize the biosynthesis of the key active constituents, thereby affecting its therapeutic efficacy.

In this study, the agricultural utility of *Priestia megaterium* HyangYak-01 as a plant growth-promoting bacterium was confirmed using plant experiments and substance analysis. The growth of *C. asiatica* treated with *P. megaterium* HyangYak-01 and production of active ingredients in its leaves exceeded that of untreated *C. asiatica*. These experimental findings suggest that the application of plant growth-promoting bacteria in sustainable and ecofriendly cultivation processes can significantly enhance the yield of medicinal herbs and increase the production of valuable bioactive compounds.

## 2. Results

### 2.1. Field Experiment Results

**Figure 1:**
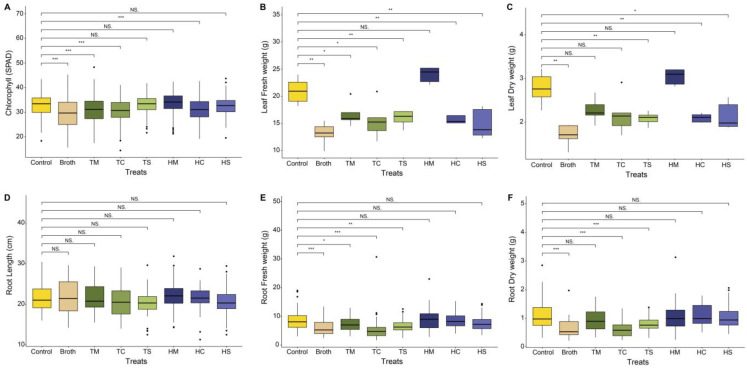
Phenotypical parameters of *C. asiatica* across the different experimental groups. (**A**) Chlorophyll content, (**B**) leaf fresh weight, (**C**) leaf dry weight, (**D**) root length, (**E**) root fresh weight, and (**F**) root dry weight. Student’s *t*-test was used for statistical calculations; NS *p* > 0.05, * *p* ≤ 0.05, ** *p* ≤ 0.01, and *** *p* ≤ 0.001.

Phenotypical parameters were measured and compared among treatments to evaluate plant growth-promoting activity. Each experimental group was named as below (Table 1). Regarding leaf-related parameters, the treatment groups, except the HM group, generally yielded lower values than the blank group across all parameters. In contrast, the HM group was the only group to generate higher values than the blank group, indicating the increased growth rate of *C. asiatica* (Figure 1A–C). In terms of root-related parameters, no significant difference was observed in root length (Figure 1D); moreover, leaf and root fresh and dry weights exhibited a consistent tendency (Figure 1E,F). Based on these results, the HM and blank groups were selected for further analysis, including metabolomics.

### 2.2. Untargeted Metabolomic Analysis of C. asiatica Leaf Metabolome

**Figure 2:**
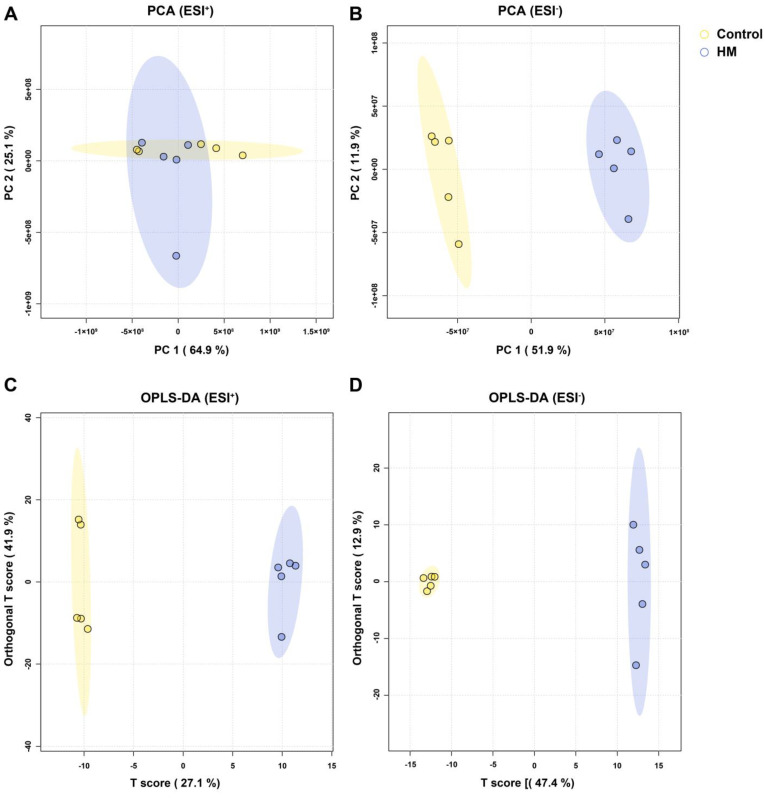
Untargeted metabolomic analysis results. Principal component analysis results of (**A**) ESI^+^ and (**B**) ESI^−^ and orthogonal partial least squares discriminant analysis results of (**C**) ESI^+^ and (**D**) ESI^−^.

**Figure 3:**
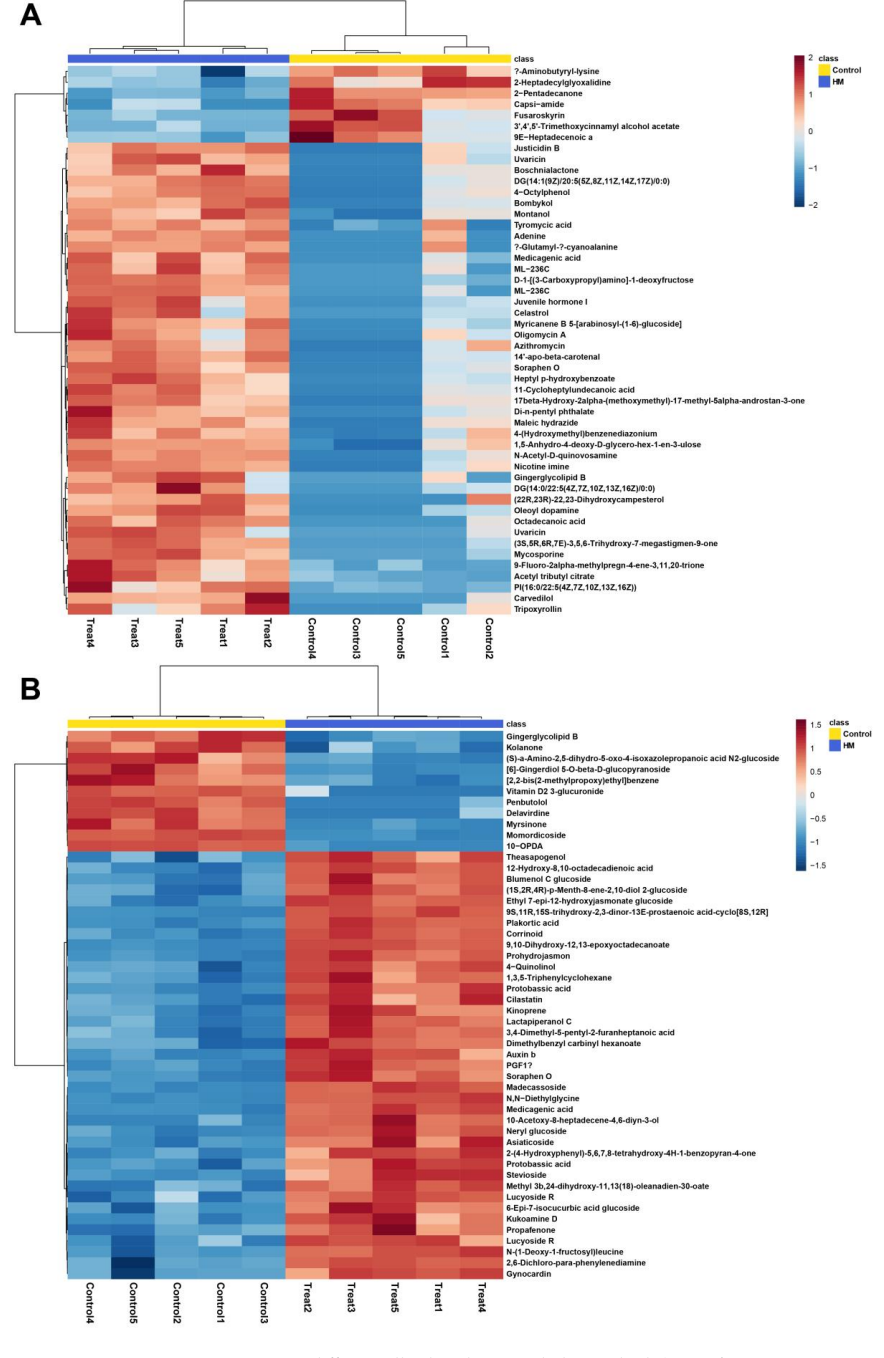
Top 50 differentially abundant metabolites in both (**A**) ESI^+^ and (**B**) ESI^−^ modes.

To confirm the leaf metabolome shift induced by treatment with the plant growth-promoting rhizobacterium (PGPR), untargeted metabolomic analysis was applied. Principal component analysis (PCA) and orthogonal partial least squares discriminant analysis (OPLS-DA) were performed to screen for treatment-induced metabolome shift in *C. asiatica* leaf samples. The results revealed a clear difference between the blank and HM groups (Figure 2). Notably, the distance was greater in ESI^−^ mode than in ESI^+^ mode. Additional analysis was conducted to determine the metabolites contributing to this difference. The 50 most differentially abundant metabolites in both ESI^+^ and ESI^−^ modes are shown in the heatmap (Figure 3). Notably, madecassoside, which is one of the most important active compounds in *C. asiatica* leaf, showed higher abundance in HM groups than the control in ESI^-^ mode. By comprehensively interpreting these metabolome data, it becomes evident that the application of *P. megaterium* HyangYak-01 induced a shift in the metabolome composition of *C. asiatica* leaves.

### 2.3. Relative Quantification of Targeted Compounds

**Figure 4:**
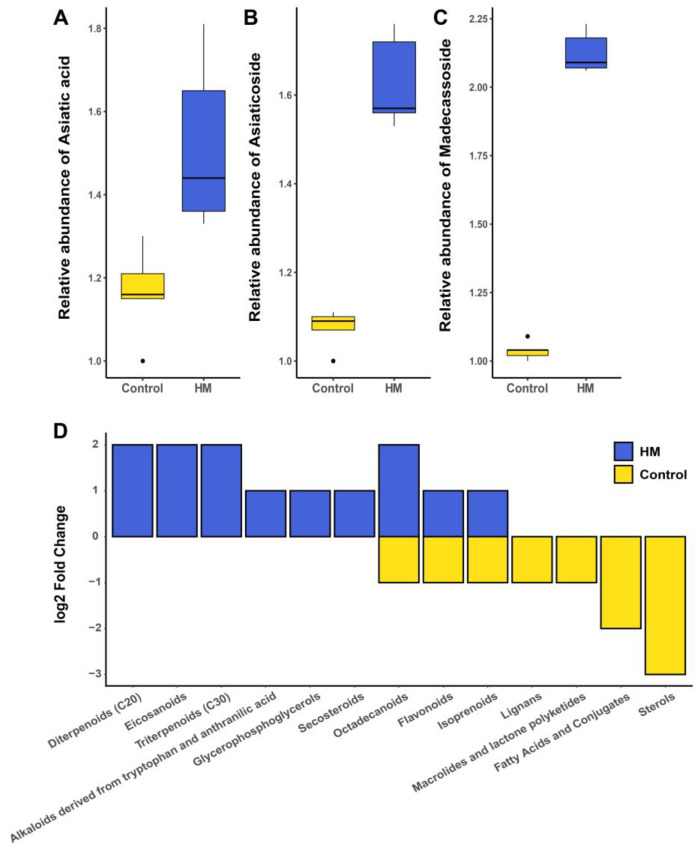
Relative quantification of main bioactive compounds and fold change values of categorized metabolites. Quantification results for asiatic acid (**A**), asiaticoside (**B**), and madecassoside (**C**). Metabolites were classified by their class and compared between groups (**D**).

Four terpenoids—madecassoside, asiaticoside, madecassic acid, and asiatic acid—were identified as the most prominent bioactive compounds in *C. asiatica* leaves. Metabolome analysis using Q-TOF-MS detected three of these four targeted active compounds. A comparative analysis of their relative abundance revealed significantly higher levels of all three compounds in the bacteria-treated group compared to the control group (Figure 4A–C).

Subsequently, detected metabolites were classified according to their chemical class, and fold change values were compared between treatment and control groups (Figure 4D). The results demonstrated that the treated group exhibited higher fold change values for triterpenoids, which include the aforementioned valuable active compounds, as well as diterpenoids, relative to the control group.

A comprehensive analysis of these results confirms that the *P. megaterium* HyangYak-01-induced shift in leaf metabolome resulted in a significant increase in the content of important bioactive compounds in *C. asiatica* leaves. This observation suggests that the application of *P. megaterium* HyangYak-01 as a plant growth-promoting rhizobacterium (PGPR) may enhance the production of therapeutically relevant compounds in *C. asiatica*.

The upregulation of terpenoid biosynthesis in response to PGPR treatment aligns with previous studies demonstrating the microbial influence on plant secondary metabolism [28]. This metabolic modulation could be attributed to various mechanisms, including the activation of plant defense responses or alterations in nutrient uptake and allocation [29]. Further investigation into the specific pathways and regulatory mechanisms involved in this PGPR-induced metabolic shift could provide valuable insights for optimizing the production of bioactive compounds in medicinal plants.

### 2.4. Cell Treatments

**Figure 5:**
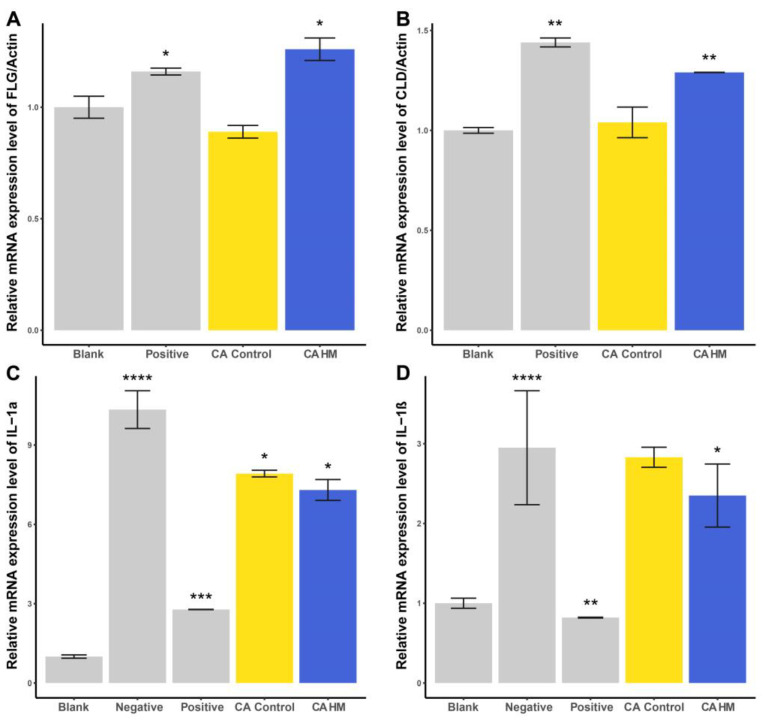
Cell experiment results following *C. asiatica* leaf extract treatment. The relative mRNA expression levels of (**A**) FLG and (**B**) CLDN1 were analyzed for their skin barrier improvement and moisturizing effects and those of (**C**) IL-1α and (**D**) IL-1β for their anti-inflammatory properties; *p* > 0.05, * *p* ≤ 0.05, ** *p* ≤ 0.01, *** *p* ≤ 0.001, and **** *p* ≤ 0.0001.

In the CA HM group, the relative mRNA expression level increased by approximately 26.2% compared with that in the blank control group. The positive control group displayed an increase in FLG/Actin expression of approximately 16.1% compared with the blank control group. The CA blank group exhibited a decrease of approximately 11.2% compared with the blank control group (Figure 5A).

In the CA HM group, the relative mRNA expression level increased by approximately 28.8% compared with that in the blank control group. The positive control group demonstrated an increase in CLDN1/Actin expression of approximately 44.2% compared with the blank control group. The CA blank group exhibited an increase of approximately 4.4% compared with the blank control group (Figure 5B).

The negative control group displayed an increase of approximately 928.5% compared with the blank control group. The positive control group exhibited an increase of approximately 177.2% compared with the blank control group. In the CA HM group, the expression level decreased by approximately 29.3% compared with that in the negative control group. The CA blank group displayed a reduction of approximately 23.3% compared with the negative control group (Figure 5C).

The negative control group demonstrated an increase of approximately 194.3% compared with the blank control group. The positive control group exhibited a decrease of approximately 72.1% compared with the blank control group. In the CA HM group, the expression level decreased by approximately 20.5% compared with that in the negative control group. The CA blank group displayed a reduction of approximately 4.0% compared with the negative control group (Figure 5D).

## 3. Discussion

The incorporation of *P. megaterium* HyangYak-01 in the cultivation of *C. asiatica* significantly enhanced the production of critical bioactive compounds, such as asiaticoside, madecassoside, asiatic acid, and madecassic acid, showcasing the profound role of PGPRs in agricultural and environmental sustainability. This study corroborates the increasing body of evidence advocating for the integration of PGPRs into mainstream agricultural practices to enhance plant metabolic processes and mitigate environmental impacts.

Our study’s results indicate that treatment with *P. megaterium* HyangYak-01 leads to a marked increase in the production of pharmacologically relevant secondary metabolites in *C. asiatica*. These metabolites, critical for their health-promoting properties, including their anti-inflammatory and wound-healing effects, were significantly more abundant in plants treated with the PGPR than in the untreated controls. These key secondary metabolites serve crucial physiological functions, such as protecting against herbivores and pathogens, regulating plant growth and development, and mediating plant–environment interactions, while also contributing to the plant’s properties [30]. This enhancement can be attributed to the PGPR’s ability to influence plant hormonal levels and upregulate the biochemical pathways involved in secondary metabolite synthesis [31]. PGPRs, including species such as *P. megaterium*, reportedly modulate the concentration of phytohormones, such as auxins, cytokinins, and gibberellins, which play pivotal roles in plant metabolism and stress responses [32,33].

Moreover, our untargeted metabolomic analyses conducted via UHPLC-QTOF-MS clearly depict the metabolic shifts induced by PGPR treatment. In this study, PCA and OPLS-DA, as illustrated in Figure 2 and Figure 3, revealed distinct clustering patterns between the treatment and control groups. These patterns provide strong evidence of a robust alteration in the plant’s metabolomic profile attributable to microbial influence [34,35]. The clear separation between clusters in both multivariate analyses underscore the significant impact of PGPR on the plant’s metabolic landscape. This pronounced metabolomic shift suggests that the PGPR treatment triggers a cascade of biochemical changes within the plant, potentially affecting various physiological processes. The observed alterations in metabolite composition not only confirm the effectiveness of the PGPR treatment but also offer insights into the complex plant–microbe interactions at the molecular level. These findings align with previous studies that have demonstrated the capacity of beneficial microorganisms to modulate plant metabolism, thereby influencing growth, development, and stress responses [36,37].

Beyond their direct benefits to plant health and yield, PGPRs such as *P. megaterium* HyangYak-01 significantly contribute to environmental sustainability. By enhancing plant growth and health, PGPRs reduce the need for chemical fertilizers and pesticides, which are major environmental pollutants. The use of these microbes potentially decreases nitrate leaching, phosphorus runoff, and pesticide residues in agricultural settings, thus mitigating the impact of agricultural effluents on terrestrial and aquatic ecosystems [38,39]. This approach not only aligns with sustainable farming practices but also promotes the resilience of agricultural systems against climate variability and soil degradation [40].

Furthermore, the economic implications of utilizing PGPRs are significant, particularly in regions reliant on agriculture as a primary economic activity. By limiting dependency on costly chemical inputs, PGPRs can lower production costs, thereby enhancing the economic viability of farming operations. This economic benefit is especially crucial for smallholder farmers in developing countries, where cost savings translate to better livelihoods and reduced poverty [41,42].

However, despite these advantages, the application of PGPRs in agriculture encounters challenges, including variability in effectiveness owing to environmental conditions and plant-specific responses. Therefore, future research should focus on customizing PGPR treatment to specific climatic and soil conditions to maximize its benefits. Studies should also explore the long-term effects of PGPRs on soil health and microbial diversity to ensure that these interventions do not inadvertently disrupt native microbial ecosystems.

## 4. Materials and Methods

### 4.1. Field Experiment and Sampling

#### 4.1.1. Experimental Design

The *C. asiatica* culture experiment was conducted at Cosmax HyangYak herb garden (11-37, Yugugyebong-gil, Yugu-eup, Gongju-si, Chungcheongnam-do, Republic of Korea), as described in a previous study. Tap water and Reasoner’s 2A (R2A) broth were treated as blank and negative control groups, respectively. *P. megaterium* KACC 10482^T^ was included to verify the effectiveness of the isolated plant growth-promoting rhizobacterium *P. megaterium* HyangYak-01 strain. To screen the effectiveness of each fraction in bacterial culture, bacterial treatments were administered via three components: the culture medium, the supernatant, and bacterial cells. They were prepared according to a previously described protocol [43]. Briefly, bacteria were inoculated in R2A media and cultured until achieving a concentration of 10^9^ CFU/mL, after which 30 mL of culture was used for treatment. Bacterial cells and the culture supernatant were separated via centrifugation for 20 min at 1300 RPM.

#### 4.1.2. Plant Material Sampling

Plants were sampled after 4 months of experimentation. Each group had 60 replicates of *C. asiatica* planted in a separate section of the field. Leaf and root samples were collected and subsequently subjected to further steps, including phenotypical measurement and metabolomic analysis. Plant phenotypical parameters comprised the following: leaf fresh and dry weights, leaf chlorophyll content, root fresh and dry weights, and length.

### 4.2. Leaf Extract Preparation

Bioactive compounds were extracted from *C. asiatica* leaves for metabolomic analysis following a previously applied protocol, with modifications [43]. Based on the phenotypical measurement results, blank and HM samples were subjected to further analysis. Collected leaves were dried for 48 h at 60 °C. Fully dried leaves were ground to a powder using a grinder and subsequently used for extraction. An amount of 1 g of each powdered sample was dissolved in 20 mL of 95% ethanol and stirred at 150 RPM and 30 °C for 48 h. After stirring, the extract was filtered using Whatman^®^ filter paper (Cytiva, MA, USA), and 5 mL of each triplicated aliquot was removed. The extract was subsequently concentrated with Eppendorf Concentrator Plus (Eppendorf, Hamburg, Germany) and resuspended in 1 mL of methanol. Thereafter, the samples were filtered using a 0.2 µm PTFE syringe filter (Korea Ace Scientific Co., Ltd., Seoul, Republic of Korea) and diluted to a final concentration of 1:100.

### 4.3. Metabolomic Analysis

#### 4.3.1. Ultra-High-Performance Liquid Chromatography-Quadrupole Time-of-Flight Mass Spectrometry (UHPLC-Q TOF-MS) Analysis

The 1290 Infinity II UHPLC and 6545XT AdvanceBio Q-TOF Systems (Agilent Technologies, Santa Clara, CA, USA), as well as the InfinityLab Poroshell 120 EC-C18 Column packed (2.7-µm, 2.1 × 150 mm, Agilent Technologies, Santa Clara, CA, USA), were used for metabolomic analysis. The mobile phase comprised water with 0.1% formic acid (A) and acetonitrile with 0.1% formic acid (B), and chromatographic separation was performed at 40 °C. The chromatographic gradient was set as follows: 0–10 min, 0–40% B; 10–18 min, 40–95%; 18–27 min, 95% B; 27–27.5 min, 95–0% B; and 27.5–30 min, 0% B. Five microliters of sample were injected, and the flow rate was set at 0.4 mL/min. Electrospray ionization (ESI) was selected as the ionization source for QTOF-MS analysis.

#### 4.3.2. Metabolite Identification and Statistical Analysis

Raw data from UHPLC-QTOF-MS were analyzed using analysis softwares. First, features were extracted from the raw data using Agilent MassHunter Profinder software 10.0. Normalization and library-based metabolite annotation were performed using Mass Profiler Professional software and MassHunter METLIN Metabolite Personal Compound Databases and Libraries. Annotated metabolome data were then statistically analyzed using MetaboAnalyst 6.0 and finally visualized using R (version 4.3.1).

### 4.4. Cell Culture and Sample Treatment

To validate the safety and effectiveness of the bacterium-treated *C. asiatica* leaf extract for application in cosmetics, cell experiments were conducted. Cell treatment and analysis were performed at Cosmax (Cosmax BTI, Inc., Seongnam-si, Republic of Korea). In vitro efficacy was analyzed using the human keratinocyte cell line HaCaT (American Type Culture Collection, Manassas, VA, USA). Cells were cultured in high-glucose Dulbecco’s modified Eagle medium (DMEM; HyClone, Logan, UT, USA) supplemented with 10% fetal bovine serum (FBS; HyClone, Logan, UT, USA) and a 1% antibiotic/antimycotic solution (Welgene, Daegu, Republic of Korea). HaCaT cells were seeded in a six-well plate with culture medium and stabilized overnight. The culture medium was replaced with FBS-free medium before sample treatment. Samples were treated at a concentration of 1%, and 1 μM retinoic acid (Sigma Aldrich, Burlington, MA, USA) was treated as the positive control group. After 24 h of culture, cells were collected for mRNA expression analysis.

For the anti-inflammatory assay, FBS-free DMEM containing 10 μg/mL of poly(I:C) HMW (InvivoGen, San Diego, CA, USA) and 10 ng/mL of recombinant human interleukin (IL)-4 protein (R&D Systems, Minneapolis, MN, USA) was administered to cells to induce inflammation. Simultaneously, 1 μM of the anti-inflammatory drug dexamethasone (Sigma Aldrich, St. Louis, MO, USA) was treated as the positive control, and 1% samples were also treated. Cell RNA was collected 4 h after treatment.

The cell RNA was isolated using the RNeasy Mini Kit (Qiagen, Valencia, CA, USA). After isolation, 1 µg of RNA was measured and converted to complementary DNA (cDNA) using ReverTra Ace™ qPCR RT Master Mix (Toyobo Co., Ltd., Osaka, Japan). Real-time polymerase chain reaction (PCR) analysis was performed using Power SYBR^®^ Green PCR Master Mix (Applied Biosystems, Warrington, UK). The cDNA was amplified with primers under the following conditions: 50 °C for 2 min, 95 °C for 10 min, 40 cycles of 95 °C for 10 s, and 60 °C for 1 min. Result data were analyzed using StepOnePlus™ software version 2.3 (Applied Biosystems, Waltham, MA, USA). Beta actin was used as an internal control, and the mRNA expression levels of the aquaporin-3 (*AQP3*), hyaluronan synthase-3 (*HAS3*), filaggrin (*FLG*), and claudin-1 (*CLDN1*) genes were calculated as relative values against the negative control group. The primer pairs used are listed in Table 2.

## 5. Conclusions

This research on the application of the PGPR *P. megaterium* HyangYak-01 in the cultivation of *C. asiatica* highlights a pivotal advancement in the field of sustainable agriculture. By significantly enhancing the production of key bioactive compounds in *C. asiatica*, this study illuminates the potential of PGPRs to bolster plant growth and metabolite synthesis, offering dual advantages of agricultural productivity and ecological sustainability.

Our findings demonstrate that PGPR treatment leads to marked increases in the concentrations of asiaticoside, madecassoside, asiatic acid, and madecassic acid, compounds known for their significant pharmacological properties. Metabolomic analyses evidently indicated that PGPR application initiates a profound alteration in the plant’s metabolic profile, promoting the synthesis of valuable secondary metabolites. These enhancements are of considerable importance not only in the pharmaceutical and cosmetic industries but also in the health sector, considering the therapeutic properties of these compounds. Nonetheless, the exact mode of action of PGPR treatment, which resulted in increased active compounds in *C. asiatica*, remains elusive. Further studies aiming to address this issue would potentially enhance its depth and impact.

Furthermore, the use of PGPRs such as *P. megaterium* HyangYak-01 aligns with environmental conservation efforts by easing reliance on chemical fertilizers and pesticides, thereby alleviating the associated ecological damage, such as soil degradation, water pollution, and biodiversity loss. This approach contributes to the sustainability of agricultural practices by enhancing soil fertility and promoting a balanced ecosystem.

## Figures and Tables

**Table 1:** Experimental group.

Treatment		Group Name
Control	Tap water	Blank
	R2A broth	Broth
*P. megaterium* KACC 10482^T^	Culture medium	TM
	Bacterial cells	TC
	Culture supernatant	TS
*P. megaterium* HyangYak-01	Culture medium	HM
	Bacterial cells	HC
	Culture supernatant	HS

**Table 2:** Real-time PCR primer sequence information.

Target	Forward	Reverse
Beta actin	5′-CATGTACGTTGCTATCCAGGC-3′	5′-CTCCTTAATGTCACGCACGAT-3′
AQP3	5′-AGACAGCCCCTTCAGGATTT-3′	5′-TCCCTTGCCCTGAATATCTG-3′
HAS3	5′-CTTAAGGGTTGCTTGCTTGC-3′	5′-GTTCGTGGGAGATGAAGGAA-3′
FLG	5′-TGAAGCCTATGACACCACTGA-3′	5′-TCCCCTACGCTTTCTTGTCCT-3′
CLDN1	5′-CTGTCATTGGGGGTGCGATA-3′	5′-CTGACCAAATTCGTACCTGGC-3′
IL-1α	5′-TGGCTCATTTTCCCTCAAAAGTTG-3′	5′-AGAAATCGTGAAATCCGAAGTCAAG-3′
IL-1β	5′-GTCATTCGCTCCCACATTCT-3′	5′-ACTTCTTGCCCCCTTTGAAT-3′

## Data Availability

Data is contained within the article and Supplementary Materials.

## Supplementary Materials

The following supporting information can be downloaded at https://www.mdpi.com/article/10.3390/plants13182636/s1.
