# Supplementary figures and images for: Metabolome Shift in Centella asiatica Leaves Induced by the Novel Plant Growth-Promoting Rhizobacterium, Priestia megaterium HyangYak-01
Source: Plants (Basel). 2024 Sep 21;13(18):2636. doi: 10.3390/plants13182636 (PMC11435292; doi:10.3390/plants13182636)

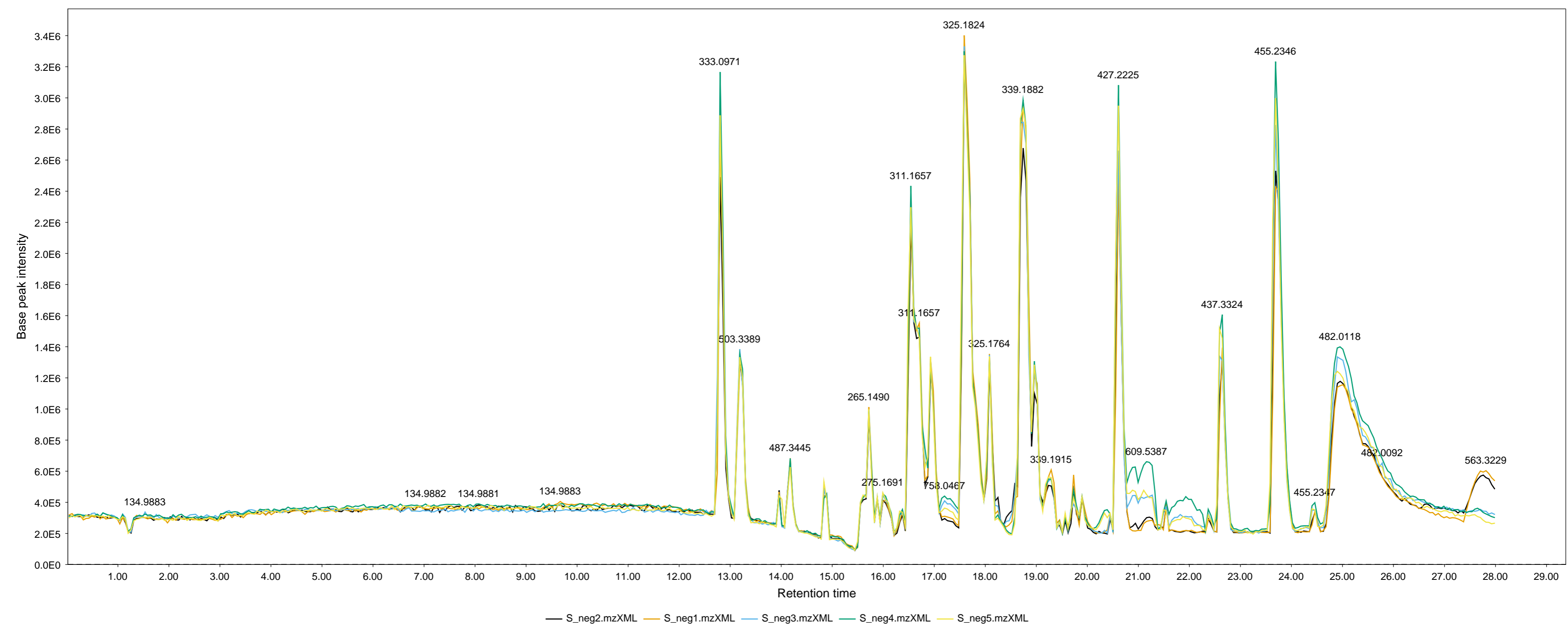

Supplement: Supplementary file 1 [file plants-13-02636-s001.zip › Control_neg.PDF]

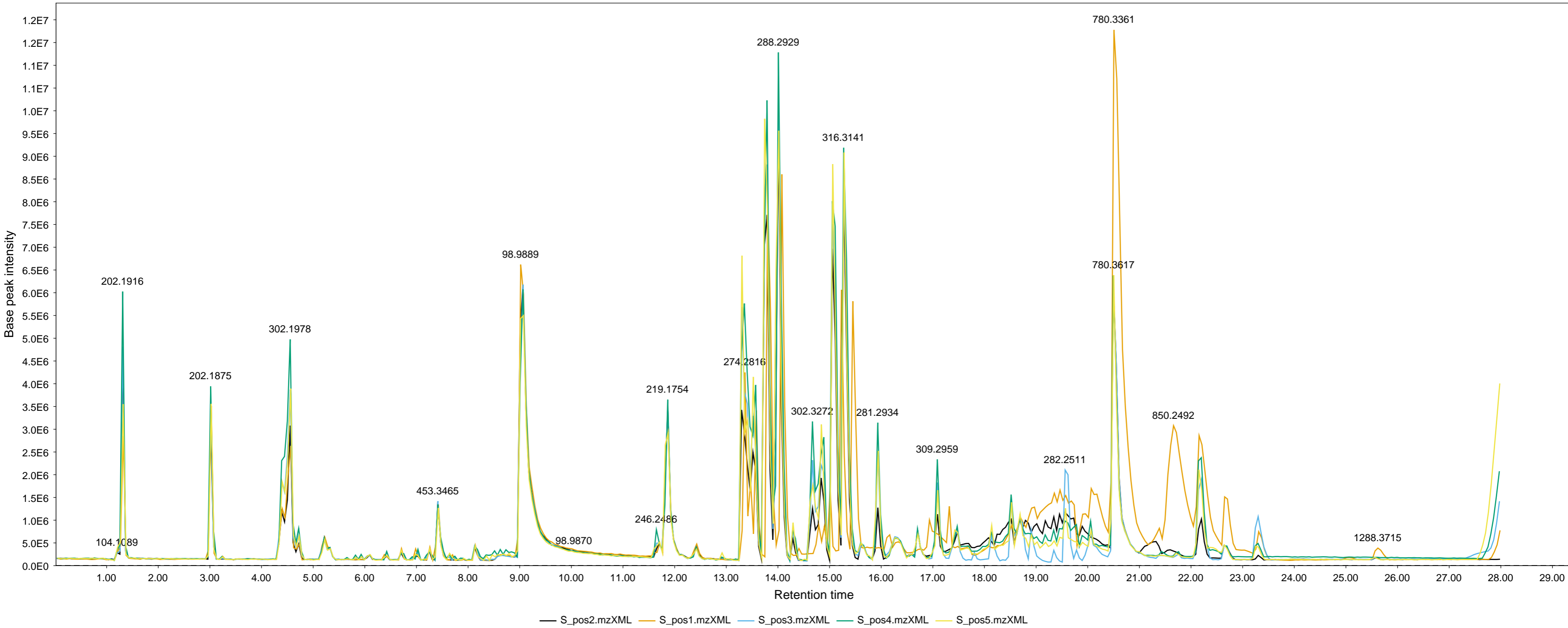

Supplement: Supplementary file 1 [file plants-13-02636-s001.zip › Control_pos.PDF]

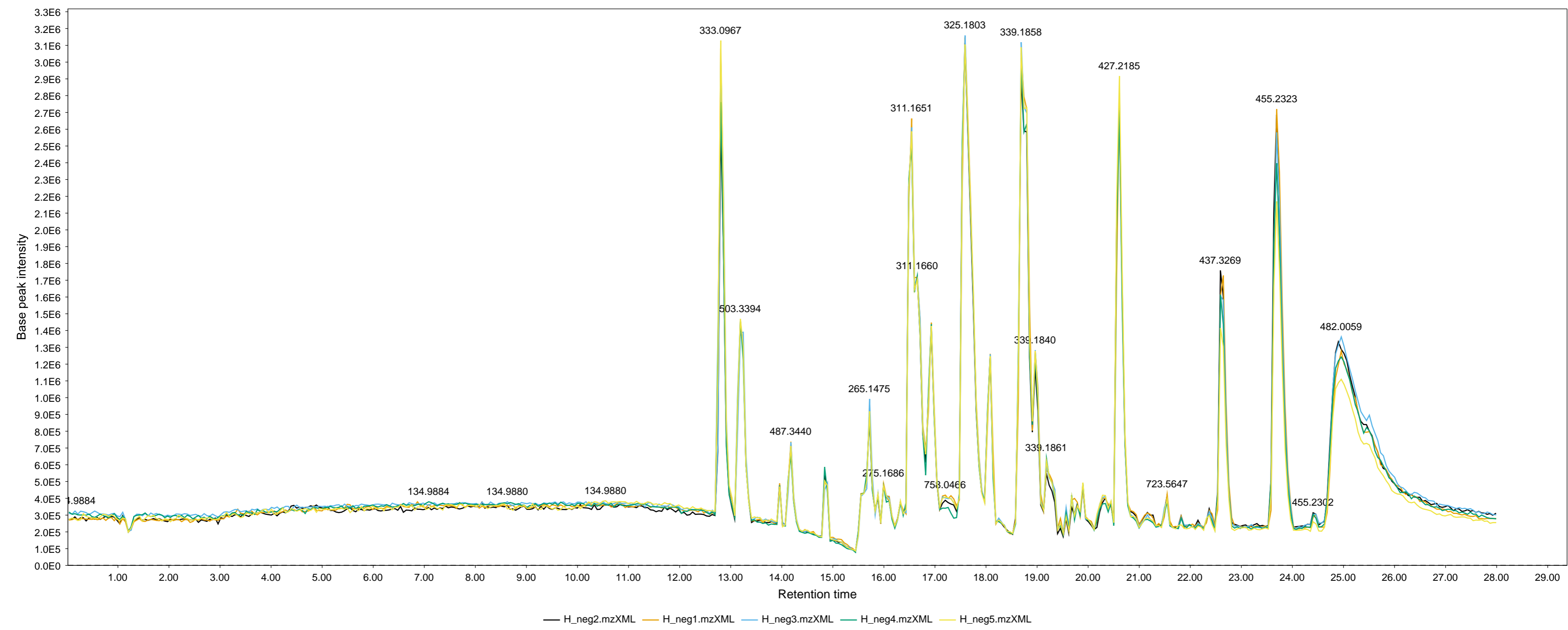

Supplement: Supplementary file 1 [file plants-13-02636-s001.zip › HM_neg.PDF]

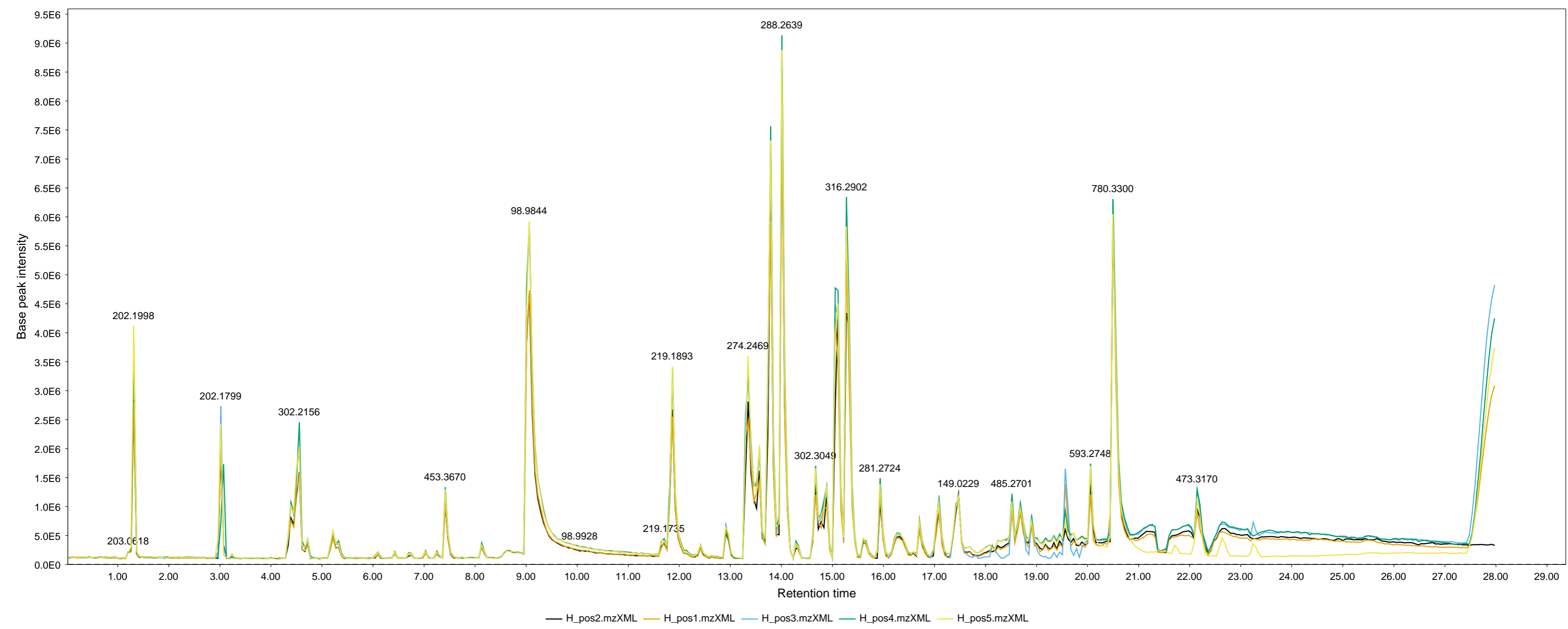

Supplement: Supplementary file 1 [file plants-13-02636-s001.zip › HM_pos.PDF]
